# Shoulder Muscle Tone, Function, and Factors Associated with Functional Recovery in Patients with Chronic Shoulder Pain: A Non-Randomized Comparative Study of Manual Therapy and Corticosteroid Injection

**DOI:** 10.3390/jcm15207854

**Published:** 2026-10-12

**Authors:** Yongtae Eom, Youngsook Bae

**Affiliations:** 1 Department of Physical Therapy, Graduate School of Public Health, Gachon University, Incheon 21936, Republic of Korea; 2 Department of Physical Therapy, College of Medical Science, Gachon University, 191 Hambangmoe-ro, Yeonsu-gu, Incheon 21936, Republic of Korea

**Keywords:** chronic shoulder pain, manual therapy, muscle tone, EQ-5D-5L, EQ VAS, posterior deltoid muscle

## Abstract

**Background/Objectives**: This study aimed to compare the effects of manual therapy (MT) and corticosteroid injection (CI) on shoulder muscle tone, function, and health-related quality of life (HRQOL) in patients with chronic shoulder pain (CSP) and to identify the muscle-tone change most strongly associated with functional recovery. **Methods**: In this quasi-experimental, non-randomized study, 44 patients with CSP lasting >6 months were allocated to MT (*n* = 22) or CI (*n* = 22) according to the treating physician’s judgment. Each group received the assigned intervention for 4 weeks, together with conventional physical therapy and a home-based exercise program. Muscle tone (upper trapezius, anterior deltoid, posterior deltoid, and latissimus dorsi [LD]), the Patient-Specific Functional Scale (PSFS), and HRQOL were assessed at the baseline and at 2 and 4 weeks. Exploratory linear regression analyses examined factors associated with changes in PSFS scores. **Results**: Both groups showed significant improvements in muscle tone, shoulder function, and HRQOL. Compared with CI, MT produced greater improvements in muscle tone, PSFS scores, and EuroQol Visual Analogue Scale (EQ VAS) scores. All participants achieved the PSFS minimal important change of ≥2 points. The proportion of participants achieving a post hoc exploratory threshold of ≥5 points was higher with MT than with CI (90.9% vs. 13.6%). In separate regression models adjusted for treatment group, changes in LD tone and EQ VAS were each associated with the change in PSFS score. **Conclusions**: Both groups showed short-term improvements, with greater gains in muscle-tone measures and function in the MT group. These findings do not establish that MT itself is superior to CI, and the exploratory LD association requires confirmation.

## 1. Introduction

Chronic shoulder pain (CSP) is among the most common musculoskeletal pain conditions, with an estimated annual prevalence of approximately 16% [1]. It represents a heterogeneous clinical entity arising from multiple etiologies, including rotator cuff disorders, adhesive capsulitis, shoulder instability, and degenerative joint disease of the shoulder [2]. Individuals whose shoulder pain persists for more than 6 months frequently experience substantial functional limitations accompanied by deterioration in psychosocial quality of life [3]. 

Current management recommendations for CSP emphasize non-surgical approaches, particularly exercise-based programs [4,5]. In clinical practice, several adjunctive treatment modalities, including manual therapy (MT) and corticosteroid injection (CI), are widely used to manage CSP. Previous studies have demonstrated beneficial effects associated with adding MT to exercise treatment [6], as well as with the use of CI [7], in patients with shoulder pain. MT has been reported to improve active and passive shoulder mobility and to reduce pain intensity. However, its effects on functional outcomes and health-related quality of life (HRQOL) remain inconclusive [8]. In contrast, CI is frequently administered during early clinical encounters to provide rapid symptom relief and facilitate short-term functional improvement [9]. 

Patients with shoulder pain have been shown to exhibit significantly increased muscle tone in the upper trapezius (UT), anterior deltoid (AD), and latissimus dorsi (LD) muscles [10]. Increased muscle tone is closely associated with pain, and musculoskeletal pain conditions are often characterized by persistent muscle activation even at rest, reflecting impaired regulation of active muscle tone [11]. Therefore, the modulation of muscle tone may represent a clinically important therapeutic target in patients with CSP, not only for pain reduction, but also for improving neuromuscular coordination and functional performance [12]. Despite the widespread clinical use of these interventions, the physiological mechanisms through which they influence muscle tone remain incompletely understood. A better understanding of how these interventions influence muscle tone is therefore needed to elucidate their mechanistic basis and to optimize their clinical application and therapeutic efficacy [13,14]. Moreover, shoulder pain may adversely affect physical function and HRQOL in patients undergoing treatment [15]. 

The relationship between changes in muscle-tone measures and improvements in shoulder function and HRQOL in patients with CSP remains unclear. Few studies have directly examined these associations, as previous research has focused primarily on pain reduction and range of motion [16,17]. MT and CI are both used in routine clinical care, with the treating physician selecting the treatment according to each patient’s clinical presentation. Comparing short-term outcomes following these clinical decisions may provide descriptive evidence about patients receiving the two treatment programs; however, physician-directed treatment selection may result in baseline differences between groups. Therefore, the purpose of this study was to compare changes in shoulder muscle tone, shoulder function, and HRQOL between patients receiving MT and CI who also performed home-based exercise, to describe baseline differences between the groups, and to explore which muscle-tone changes were most strongly associated with functional improvement. The between-group comparisons were interpreted as observational associations rather than causal estimates of the relative effectiveness of MT and CI.

## 2. Materials and Methods

### 2.1. Study Design and Ethics

The study was conducted in accordance with the Declaration of Helsinki and was approved by the Institutional Review Board of Gachon University (Approval No. 1044396-202501-HR-001-01; approval date: 25 February 2025). Before enrollment, all participants received a comprehensive explanation of the study objectives and procedures and provided written informed consent. 

This quasi-experimental, non-randomized comparative study allocated participants to an MT group or a CI group according to the treating physician’s clinical judgment. Randomization was not performed for ethical and clinical reasons: treatment selection was left to the treating physician to ensure patient safety and appropriate care in a real-world clinical setting. After baseline assessment, the treating physician selected MT or CI according to routine clinical judgment and the perceived suitability of each treatment for the patient. No prespecified treatment-allocation algorithm was used. The research investigators did not determine or modify the treatment decision. The specific patient-level factors underlying each decision were not systematically recorded as study variables; therefore, these factors could not be summarized by treatment group or included in adjusted analyses.

### 2.2. Participants

This study included patients with CSP who visited a hospital. Participants were eligible if they had unilateral shoulder pain lasting ≥6 months, were aged 18–75 years, and demonstrated clinical signs of impingement syndrome or rotator cuff lesions confirmed by positive Neer, Hawkins–Kennedy, and Empty Can (Jobe) tests [18,19]. Participants were excluded if they had a history of shoulder surgery; a history of allergy to the study medications; traumatic shoulder dislocation within the past 3 months; injection or MT for shoulder pain within the past 6 months; neurological symptoms involving the shoulder; systemic inflammatory joint disease; a full-thickness rotator cuff tear; any contraindications to MT of the shoulder; or were pregnant or suspected to be pregnant [19,20].

**Figure 1:**
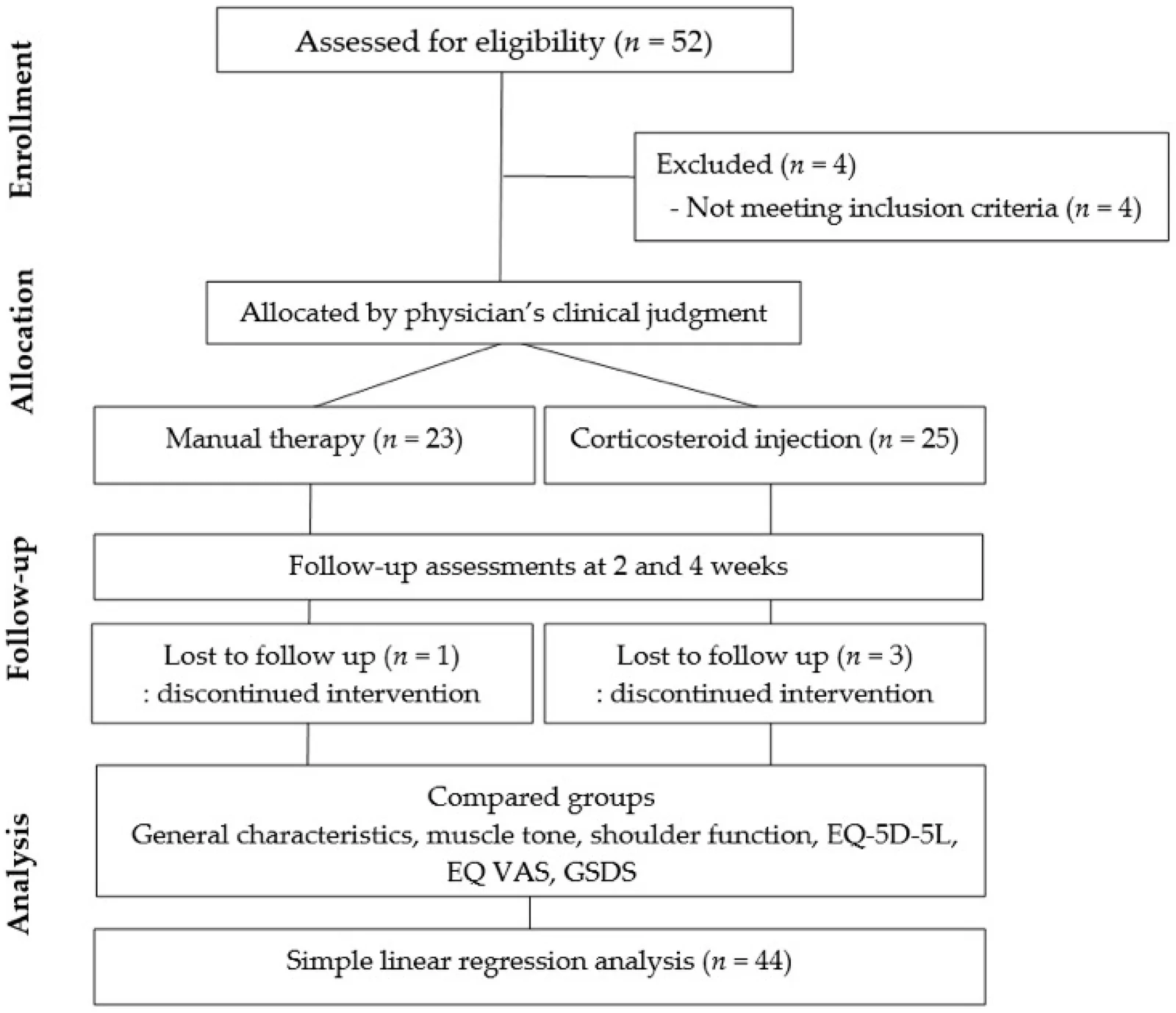
Flow diagram of the study.

The sample size was calculated using G*Power software (version 3.1.2; Franz Faul, University of Kiel, Kiel, Germany). A repeated-measures analysis of variance (ANOVA) model with two groups and three measurement time points was applied to test within-group, between-group, and group × time interaction effects. Assuming a medium effect size (f = 0.25), a significance level (α) of 0.05, and a statistical power (1 − β) of 0.95, the minimum required sample size was 44 participants. Of the 52 participants initially screened for eligibility, four did not meet the inclusion criteria, so 48 were enrolled in the study. During the intervention period, several participants in both groups were excluded because of protocol violations or voluntary withdrawal, resulting in 22 participants remaining in each group. Consequently, 44 participants were included in the final analysis (Figure 1). Both groups received the same 30-min conventional physical therapy session consisting of 15 min of interferential current (IFC) therapy and 15 min of low-level laser therapy (LLLT).

### 2.3. Procedures and Interventions

The study was conducted between March and May 2025, and all data were collected in a hospital setting. Baseline assessments were completed before treatment selection. Participants were assigned to the MT or CI group according to the treating physician’s clinical judgment, which took into account clinical characteristics and the appropriateness of each treatment. Group allocation was conducted by an investigator who was not involved in participant recruitment or outcome assessment to minimize potential allocation bias. During the 4-week intervention period, participants in the MT group received one supervised manual therapy session per week, resulting in four MT sessions. Participants in the CI group received one fluoroscopy-guided intra-articular corticosteroid injection per week, resulting in four injections. Both groups received conventional physical therapy on the scheduled treatment day and performed the standardized home exercise program 6 days per week. Outcome measures were assessed at the baseline (pre-intervention) and at 2 and 4 weeks following the intervention.

#### 2.3.1. Manual Therapy 

MT was administered by a licensed physical therapist with more than 6 years of clinical experience in musculoskeletal rehabilitation. Each MT session lasted approximately 40 min and was delivered according to a standardized treatment protocol to ensure consistency across participants. The intervention consisted primarily of joint mobilization techniques targeting the shoulder complex and scapulothoracic articulation. These techniques included general joint mobilization; superior-to-inferior gliding to facilitate shoulder flexion and abduction; anterior-to-posterior and posterior-to-anterior glides to improve joint play; and mobilization techniques targeting shoulder internal and external rotation. In addition, scapular mobilization techniques focusing on internal and external rotation of the scapula were performed to enhance scapulohumeral rhythm. The intensity of mobilization was individually adjusted according to each participant’s pain tolerance and clinical presentation, corresponding to Kaltenborn Grades II–III traction and gliding techniques, with the primary therapeutic goals of reducing pain and improving joint mobility [21,22,23]. 

#### 2.3.2. Corticosteroid Injection 

CI was administered by a pain medicine specialist with more than 10 years of clinical experience under fluoroscopic guidance using a C-arm imaging system. Participants were placed in the supine position, and anatomical landmarks, including the posterior margin of the acromion and the humeral head, were palpated while the shoulder was maintained in external rotation to facilitate identification of the glenohumeral joint line. After the needle reached the joint capsule, a contrast medium was injected to confirm accurate intra-articular placement, and fluoroscopic images were obtained. Correct needle placement was verified by uniform intra-capsular distribution of the contrast agent [24,25]. Previous studies administered intra-articular triamcinolone at 20 mg per injection on four occasions over approximately 1 month [26] or at 40 mg per injection once weekly for 3 weeks [27]. In the present study, each participant in the CI group received four weekly fluoroscopy-guided intra-articular injections, each containing 10 mg of triamcinolone and 10 mL of 0.3% lidocaine, for a cumulative triamcinolone dose of 40 mg. This regimen reflects a dose commonly used for intra-articular corticosteroid injection in shoulder disorders, balancing anti-inflammatory efficacy against the risk of steroid-related adverse effects.

#### 2.3.3. Conventional Physical Therapy 

After MT or CI, both groups received adjunctive IFC and LLLT for pain relief, with each modality applied for 15 min under identical conditions [28,29]. IFC was delivered using a medium-frequency electrical stimulation device (GP-MEDPLUS, Goodple, Wonju-si, Republic of Korea), which applied alternating currents at a carrier frequency of 4000–4100 Hz to the affected shoulder region for 15 min. The stimulation intensity was adjusted to a level that each participant found comfortable, and the suction force was set to the minimum level required to maintain adequate electrode attachment to the skin. Electrodes were positioned to cover the anterior, posterior, lateral, and superior aspects of the shoulder joint to provide comprehensive stimulation of the affected region. 

LLLT was administered using a scanning laser device (Scan-Laser HLA-200, Juwon Medical, Gyeongsan-si, Republic of Korea) with a wavelength of 600 nm and an output power of 30–60 mW. The therapy was applied continuously to the affected shoulder region for a total of 15 min and was performed immediately after IFC. 

#### 2.3.4. Home Exercise Program 

All participants were instructed to perform a standardized home-based shoulder activation exercise program throughout the intervention period. The program consisted of two isometric exercises designed to facilitate activation of the shoulder and scapular musculature. The first exercise involved pulling against a towel while maintaining the elbows flexed at 90° and keeping the upper arms close to the trunk, thereby minimizing compensatory shoulder movement. The second exercise required participants to pull against the towel with the shoulder flexed to 90° and the elbows fully extended, with the aim of activating the shoulder and scapular muscles in an elevated arm position. Each isometric contraction was maintained for 1 min per set and repeated for five sets, with a 20-s rest interval between sets, resulting in a total exercise duration of approximately 10 min per session. Participants were instructed to avoid shoulder shrugging and excessive trunk movement and were encouraged to concentrate on perceiving muscle activation in the shoulder and scapular region to ensure appropriate neuromuscular engagement. Participants were instructed to perform the home-based exercise program 6 days a week. Adherence was monitored and confirmed through weekly mobile questionnaires and a digital self-reporting system, with automated reminders sent when reports were missing. All participants reported completing the prescribed exercises on every scheduled home exercise day during the 4-week intervention period.

MT, CI, and conventional physical therapy were administered once weekly for a total of 4 weeks. To ensure consistency in treatment scheduling across participants, all interventions were delivered on the same day of the week. The home exercise program was performed 6 days a week for 4 weeks, excluding the days on which MT and CI were administered.

### 2.4. Outcome Measures

The primary outcomes of this study were shoulder muscle tone and shoulder function, while HRQOL was a secondary outcome.

#### 2.4.1. Muscle Tone

Shoulder muscle tone was evaluated by measuring the muscle tone frequency (Hz) of the upper trapezius (UT), anterior deltoid (AD), posterior deltoid (PD), and latissimus dorsi (LD) using a MyotonPRO device (Myoton AS, Tallinn, Estonia). These muscles were selected because of their key roles in shoulder stability and scapulohumeral movement.

Measurement sites were standardized to ensure consistency across participants. For the UT, the measurement point was defined as the midpoint between the acromion and the spinous process of the seventh cervical vertebra [30]. For the AD, the midpoint between the clavicle and the deltoid tuberosity was used. For the PD, the measurement site was located approximately two fingerbreadths posterior to the posterior border of the acromion. For the LD, the measurement point was defined as 5 cm superior to the inferior angle of the scapula, with the participant positioned supine and the shoulder flexed to 90° [10]. All measurements were performed while the muscles were maintained in a fully relaxed state. The MyotonPRO device quantifies muscle mechanical properties by applying a brief mechanical impulse to the subcutaneous tissue overlying the target muscle or tendon under a constant pre-load of 0.18 N. The resulting oscillations within the underlying tissue were detected by the probe and analyzed to calculate viscoelastic parameters. The MyotonPRO device was operated in multi-scan mode, with measurement parameters set to three mechanical taps and a tap duration of 15 ms. To minimize measurement error, the mean of three repeated measurements at each site was used for statistical analysis [31]. The reliability of Myoton-derived measurements has been reported to be high in previous studies [32]. 

#### 2.4.2. Shoulder Function

Shoulder function was assessed using the Patient-Specific Functional Scale (PSFS). The PSFS is a reliable, valid, and responsive outcome measure for assessing functional change in patients with shoulder pain, with good construct validity for evaluating upper-limb functional limitations and strong responsiveness to change over time [33]. At the baseline, participants were asked to identify up to five activities that were difficult or impossible to perform because of their shoulder condition. Examples of nominated activities included reaching overhead, dressing, lifting objects, and sleeping on the affected side. Each selected activity was rated using an 11-point numeric rating scale ranging from 0 (“unable to perform the activity”) to 10 (“able to perform the activity at the pre-injury level”). The same activities were re-rated at each follow-up assessment to quantify within-patient functional changes over time. The PSFS total score was calculated as the mean of the item scores (sum of item scores divided by the number of activities rated), with higher scores indicating better functional status. When fewer than five activities were nominated, the mean score was calculated using only the available items.

#### 2.4.3. Health-Related Quality of Life

HRQOL was assessed using the EuroQol 5-Dimension 5-Level questionnaire (EQ-5D-5L), the EuroQol Visual Analogue Scale (EQ VAS), and a sleep disturbance measure.

The Korean version of the EQ-5D-5L used in this study has previously been validated [34]. Compared with the three-level (3L) version, the EQ-5D-5L demonstrates superior validity at both the individual and group levels, with greater sensitivity and measurement precision across a wide range of health conditions and substantially reduced ceiling effects [35]. Health utility values range from 1 (full health) to 0 (death) and are derived using a country-specific value set that assigns preference-based weights to health states defined by the EQ-5D-5L classification system [36]. The EQ-5D-5L consists of a descriptive system comprising five dimensions: mobility, self-care, usual activities, pain/discomfort, and anxiety/depression. The combination of five dimensions and five response levels generates 3125 distinct health states, with the health state coded as 11,111 representing full health and 55,555 representing the poorest health status. Each health state is identified using a five-digit code, in which each digit corresponds to the level reported for one dimension. The EQ-5D-5L Index score was calculated by applying the EQ-5D-5L scoring algorithm, with a lower score indicating a poorer quality of life [34].

The EQ VAS is a self-reported instrument in which patients rate their overall health status by marking a position on a vertical visual analogue scale. The scale is bipolar, ranging from 0 (“the worst health you can imagine”) to 100 (“the best health you can imagine”). The EQ VAS provides a quantitative measure of health outcomes that reflects patients’ subjective perceptions of their overall health status. The EQ VAS has been shown to be reliable for assessing HRQOL in this population [37]. 

In patients with shoulder pain, poor sleep quality has been associated with lower EQ-5D Index scores, suggesting a close relationship between sleep disturbance and HRQOL [38]. Accordingly, sleep disturbance was assessed using the General Sleep Disturbance Scale (GSDS), which evaluates sleep-related problems experienced over the previous 7 days. The GSDS consists of 21 items organized into seven subscales: difficulty initiating sleep (1 item), nighttime awakenings (1 item), early morning awakening (1 item), sleep quality (3 items), sleep quantity (2 items), daytime fatigue and alertness at work (7 items), and use of sleep-promoting medications (6 items). Each item is rated on an 8-point Likert scale ranging from 0 (never) to 7 (every day). After reverse scoring items 4, 10, and 11, total scores were calculated by summing all items (range: 0–147), with scores ≥ 43 indicating general sleep disturbance [39]. The Korean version of the GSDS has been validated and shown to be reliable for detecting sleep disturbance [40].

### 2.5. Statistical Analysis

Statistical analyses were performed using SPSS Statistics for Windows, version 26.0 (IBM Corp., Armonk, NY, USA). Descriptive statistics were calculated to summarize participant demographic characteristics and baseline measurements. Baseline differences between the MT and CI groups were described using standardized mean differences (SMDs). The normality of continuous variables was assessed using the Shapiro–Wilk test. Within-group changes in outcome variables measured at the baseline and at 2 and 4 weeks after the intervention were analyzed using repeated-measures ANOVA. Between-group differences in changes over time were examined using a two-way repeated-measures ANOVA model, with particular emphasis on the group × time interaction effect. Effect sizes were quantified using partial eta squared (η^2^), with values of 0.02, 0.13, and ≥0.26 indicating small, moderate, and large effects, respectively [41]. The sphericity assumption for each repeated outcome was assessed using Mauchly’s test. When sphericity was violated (*p* < 0.05), the Greenhouse–Geisser correction was applied to the degrees of freedom and *p* values for the time and group × time effects. To account for baseline outcome values in this non-randomized study, separate analysis of covariance (ANCOVA) sensitivity analyses compared the groups at week 4, adjusting each outcome for its corresponding baseline value. To explore factors associated with functional improvement, separate multiple linear regression models were fitted with the change in PSFS score from the baseline to week 4 as the dependent variable. Each model included a change in one predictor of interest (a muscle-tone measure [UT, AD, PD, or LD], the EQ-5D-5L Index, or the EQ VAS score), with treatment group as a covariate.

We first assessed the proportion of participants achieving the previously reported PSFS minimal important change (MIC) of ≥2.0 points for patients with shoulder pain [42]. Because all participants met this threshold, it did not distinguish the magnitude of improvement between groups. We therefore examined a ≥5.0-point improvement as a post hoc, exploratory threshold to describe larger functional gains. This threshold was selected for the present analysis and is not an established MIC. Responder proportions were compared using Fisher’s exact test. The principal comparative analyses examined between-group differences in outcomes at week 4 after adjustment for their corresponding baseline values. The analyses of individual muscle-tone measures and other outcomes, within-group changes, associations with PSFS change, and the post hoc ≥ 5-point PSFS responder threshold were considered secondary or exploratory. Given the exploratory scope of these analyses, no formal adjustment for multiple testing was applied; accordingly, *p* values from these analyses are reported as nominal and were interpreted alongside effect estimates and confidence intervals rather than as confirmatory evidence. Data are presented as mean ± standard deviation (SD). Statistical significance was set at α = 0.05.

## 3. Results

Baseline demographic and clinical characteristics of the participants are presented in Table 1.

Both the MT and CI groups demonstrated progressive reductions in muscle tone from the baseline to 2 and 4 weeks. The MT group showed significantly greater reductions in muscle tone over time than the CI group (group × time interaction) in the UT (*p* < 0.001, η^2^ = 0.388), AD (*p* < 0.001, η^2^ = 0.518), PD (*p* < 0.001, η^2^ = 0.575), and LD (*p* < 0.001, η^2^ = 0.309) (Table 2).

Table 3 presents shoulder function, HRQOL, and sleep disturbance in both treatment groups at the baseline and at 2 and 4 weeks. PSFS, EQ-5D-5L, and EQ VAS scores increased, whereas GSDS scores decreased over time in both groups. After adjustment for the corresponding baseline values, the MT group had higher PSFS and EQ VAS scores at 4 weeks than the CI group (adjusted mean differences, 1.43 and 10.81, respectively; both *p* < 0.001). The adjusted difference in GSDS was not statistically significant. The adjusted EQ-5D-5L score was lower in the MT group in the main analysis (*p* = 0.004), although this finding was sensitive to the baseline-adjustment model (*p* = 0.070).

Exploratory linear regression analyses showed that among the muscle-tone measures, only the change in LD tone was associated with the change in PSFS score in the model adjusted for treatment group and changes in the other muscles (β = 0.268, *p* = 0.030). Among the patient-reported measures, the change in EQ VAS showed the strongest association with the change in PSFS score (β = 0.372, *p* < 0.001), whereas the change in EQ-5D-5L was not significant (Table 4).

The clinical relevance of changes in shoulder function was examined using a threshold-based responder analysis. At week 4, all participants in both groups showed an improvement of ≥2.0 points in PSFS score, which corresponds to the previously reported MIC for patients with shoulder pain (22/22 [100%] in each group). A post hoc exploratory analysis using a more stringent threshold of ≥5.0 points showed that a significantly greater proportion of participants in the MT group than in the CI group met this threshold (20/22 [90.9%] vs. 3/22 [13.6%]; Fisher’s exact *p* < 0.001).

## 4. Discussion

This study compared the effects of MT and CI on shoulder muscle tone, shoulder function (PSFS), HRQOL (EQ-5D-5L, EQ VAS), and sleep disturbance (GSDS) in patients with CSP. Both groups also performed identical home-based exercise programs throughout the intervention period to control for the potential influence of exercise-related effects. The findings showed that both MT and CI were associated with significant improvements in muscle tone, shoulder function, and HRQOL over time. Notably, MT was associated with greater improvements than CI in shoulder muscle tone, PSFS score, and EQ VAS score.

Previous studies have shown that MT can significantly reduce muscle tone in patients with low back pain [43], whereas CI is widely recognized as an effective intervention for reducing musculoskeletal pain [44]. Furthermore, both MT and CI have been associated with improvements in pain and functional outcomes across various musculoskeletal pain conditions [45]. Changes in muscle tone may be partly mediated by alterations in somatosensory input, as mechanical stimulation of sensory afferents has been shown to induce adaptive responses in muscle spindles, potentially modulating muscle length sensitivity and resting muscle tone [46,47]. Within this context, MT may facilitate functional recovery through multiple mechanisms, including reductions in pain, tone, and fatigue; improvements in joint range of motion [48]; and alleviation of pain-related dysregulation of active muscle tone [49]. In the presence of musculoskeletal pain, the regulation of muscle tone may become disrupted, and muscle tone is closely associated with muscle excitability [50,51], which may manifest as increased pain-related protective muscle activity. In the present study, the muscle tone of the UT, AD, PD, and LD decreased progressively over time relative to the baseline. By week 4, the MT group demonstrated greater reductions in muscle tone across all four muscles than the CI group. These findings show that changes in MyotonPRO-measured muscle-tone frequency differed between the treatment groups. However, pain intensity, protective muscle activity, neuromuscular excitability, and neuromuscular coordination were not directly measured. We therefore cannot determine whether changes in these processes explain the observed differences in muscle-tone frequency or functional improvement. Future studies should assess pain intensity and relevant physiological measures alongside muscle mechanical properties and function to investigate these potential relationships.

Shoulder function, assessed using the PSFS, improved in both the MT and CI groups. The mean PSFS score improved by 5.67 points in the MT group and by 4.32 points in the CI group from the baseline to week 4. All participants in both groups exceeded the previously reported MIC of 2.0 points for patients with shoulder pain, indicating clinically important functional improvement with both treatment approaches [42]. However, because the MIC responder rate was 100% in both groups, this threshold did not distinguish between the magnitudes of improvement. In a post hoc exploratory analysis using a more stringent threshold of ≥5.0 points, a significantly greater proportion of participants in the MT group than in the CI group met the threshold (90.9% vs. 13.6%). Nevertheless, the between-group difference observed using the ≥5-point threshold should be interpreted as an exploratory, hypothesis-generating finding rather than evidence of an established clinically important difference. These functional findings should be considered within the broader evidence on rehabilitation for shoulder disorders. The 2025 clinical practice guideline for rotator cuff tendinopathy recommends individualized education and active rehabilitation exercises, including motor control and resistance training, as initial management. Manual therapy may also be considered for short-term pain relief, although its optimal techniques and dosage remain uncertain [52]. 

Among the four muscles examined, PD tone showed the largest group × time interaction effect (partial η^2^ = 0.575), indicating that its pattern of change differed most markedly between the MT and CI groups. However, PD tone change was not significantly associated with PSFS change in the exploratory regression model adjusted for treatment group and changes in the other muscles. In the same model, LD tone change was associated with PSFS change (β = 0.268, *p* = 0.030). Both PD and LD contribute to shoulder extension, but this shared anatomical role does not explain their differing statistical findings. In particular, the between-group difference in PD tone change does not establish an association between individual PD tone changes and the extent of functional improvement, while the LD association does not establish a causal role in recovery. Given the small sample size, larger studies are needed to determine whether these findings are reproducible and to clarify how changes in individual muscle tone relate to functional improvement. Sports participation is another relevant consideration when interpreting shoulder function. Chiaramonte et al. observed greater dominant-shoulder external rotation in asymptomatic adolescent volleyball players without corresponding evidence of instability, illustrating that sport-specific adaptations do not necessarily indicate pathology [53]. Although this study did not assess recovery after injury, it highlights the importance of considering activity demands when evaluating shoulder function.

In the present study, the EQ-5D-5L Index, EQ VAS, and GSDS scores improved significantly from the baseline in both the MT and CI groups. However, a significant group × time interaction favoring the MT group was observed only for the EQ VAS, whereas no significant between-group differences in changes over time were found for the EQ-5D-5L Index or GSDS scores. These findings suggest that overall health status and sleep disturbance improved in both groups, whereas the improvement in perceived overall health was greater in the MT group. The absence of a significant between-group difference in the EQ-5D-5L Index may be related to the generic nature of this instrument. The EQ-5D-5L assesses broad dimensions of health, including mobility, self-care, usual activities, pain/discomfort, and anxiety/depression, and may therefore be less responsive to short-term functional changes involving a specific anatomical region [36]. In contrast, the GSDS is a sleep-specific, multidimensional measure, and sleep disturbance may be influenced by various physical and psychosocial factors beyond shoulder function. The EQ VAS reflects an individual’s perception of their current overall health status and may be more responsive to short-term changes in symptoms and functional ability [37,54]. In an exploratory model of health-related outcomes adjusted for treatment group, the change in EQ VAS was associated with the change in PSFS score (β = 0.372, *p* < 0.001) whereas EQ-5D-5L and GSDS changes were not. Participants reporting greater improvement in patient-specific shoulder function also tended to report greater improvement in their overall health. However, because both the PSFS and EQ VAS are patient-reported measures, the strength of this association may partly reflect shared method variance [55]. This pattern suggests that functional recovery and HRQOL may improve along different trajectories and that improvements in joint-specific function may not be uniformly reflected across all domains of quality of life.

Overall, both groups showed improvements in muscle tone, shoulder function, HRQOL, and sleep disturbance during their respective 4-week multimodal treatment programs. Compared with the CI-based program, the MT-based program was associated with greater improvements in shoulder muscle tone, PSFS, and EQ VAS. In the exploratory models adjusted for treatment group, PD tone change was not associated with PSFS change, whereas LD tone change and EQ VAS change were associated with PSFS change in separate models. These associations do not establish that changes in muscle tone caused functional improvement. These analyses used pooled change scores with adjustment for treatment group and do not establish a causal role for individual muscle-tone changes in functional recovery. The association between PSFS and EQ VAS may also partly reflect shared method variance because both are patient-reported measures.

Both groups received IFC and LLLT and were prescribed home exercise in addition to their assigned MT or CI treatment. Thus, the within-group improvements cannot be attributed to MT or CI alone. The between-group findings compare two multimodal programs that differed in their assigned treatment. Because allocation was not randomized, these findings should not be interpreted as definitive evidence that MT itself caused the larger improvements.

This study has several limitations. First, treatment selection was not randomized and was based on the treating physician’s routine clinical judgment without a prespecified allocation algorithm. The patient-level reasons for selecting MT or CI and specific shoulder diagnoses were not systematically documented. Participants were enrolled based on chronic shoulder pain and compatible clinical signs rather than a standardized diagnosis of a single shoulder disorder. Consequently, we could not report the distribution of underlying shoulder conditions by treatment group or determine whether differences in diagnosis, pretreatment pain characteristics, treatment history, range of motion, imaging findings, analgesic use, work status, psychosocial characteristics, comorbidities, or patient preferences influenced treatment selection and outcomes. These factors could not be included in the adjusted analyses. Sports participation and training exposure were not recorded, preventing assessment of their potential associations with functional outcomes. Statin use was also not recorded. Recent evidence on the association between statin exposure and shoulder tendinopathy is conflicting, and observational studies and individual case reports cannot establish a causal relationship [56]. Therefore, we could not determine whether statin exposure differed between groups or was associated with the observed outcomes. Confounding by indication may have contributed to the observed between-group differences, but its direction and magnitude cannot be determined from the available data. The greater improvements observed in the MT group should not be interpreted as establishing the causal superiority of MT over CI. The small sample size and single-center setting further limit the precision and generalizability of these findings. Second, the interventions were administered only once weekly for 4 weeks, and outcomes were assessed only at the baseline and at 2 and 4 weeks. Consequently, the long-term durability of the observed improvements could not be determined. Future studies should include follow-up assessments at 3 and 6 months after treatment to evaluate the sustainability of the treatment effects more comprehensively. Third, owing to the nature of the interventions, blinding of participants and therapists was not feasible, potentially introducing performance and expectation biases. In addition, it remains unclear whether differences in home exercise adherence influenced the outcomes. Fourth, pain intensity was not assessed as a primary outcome, limiting the ability to directly examine the relationships among changes in muscle tone, pain reduction, and functional recovery. Participants may also have had heterogeneous underlying shoulder pathologies that were not considered in the analyses, potentially contributing to variability in treatment responses. Finally, the regression analyses examining factors associated with functional recovery were exploratory and based on pooled change scores. Although treatment group was included as a covariate in the regression models, no adjustment for multiple comparisons was applied. Furthermore, because both the PSFS and EQ VAS are patient-reported measures, their observed association may have been partly influenced by shared method variance. Therefore, these associations should not be interpreted as causal, and the findings should be confirmed in future randomized controlled trials with larger sample sizes and longer follow-up periods.

## 5. Conclusions

Both the MT and CI groups showed short-term improvements in shoulder muscle-tone measures, patient-specific function, and health-related outcomes during their 4-week multimodal treatment programs. Greater improvements in several muscle-tone measures and PSFS scores were observed in the MT group than in the CI group. However, non-randomized treatment selection, concomitant therapies, the small sample size, and the short follow-up period preclude a causal conclusion that MT itself is more effective than CI. In exploratory analyses adjusted for treatment group, PD tone change was not associated with PSFS change, whereas LD tone change and EQ VAS change were associated with PSFS change in separate models. These associations do not establish mechanisms of functional recovery and require confirmation in larger studies. 

## Figures and Tables

**Table 1:** Baseline characteristics of the participants.

	Manual Therapy (n = 22)	Corticosteroid Injection (n = 22)	*p*
Sex (male/female)	6/16	7/15	0.741 *
Age (years)	47.18 ± 10.14	47.64 ± 8.98	0.876 ^†^
Height (cm)	163.09 ± 7.40	165.82 ± 6.93	0.214 ^†^
Weight (kg)	59.77 ± 11.30	60.09 ± 9.66	0.921 ^†^
Onset time (month)	10.31 ± 3.09	9.90 ± 2.77	0.124 ^†^
Affected side (left/right)	12/10	16/6	0.210 *

* Statistical analysis was performed using the chi-square test; ^†^ Statistical analysis was performed using the independent *t*-test. Values are expressed as mean ± standard deviation.

**Table 2:** Change in muscle tone before and after the intervention.

	Manual Therapy				Injection Therapy				Group × time		
	Baseline	2 Weeks	4 Weeks	*p* (η^2^)	Baseline	2 Weeks	4 Weeks	*p* (η^2^)	F	*p*	η^2^
UT	19.69 ± 2.05	18.52 ± 1.83	17.56 ± 1.72 ^abc^	<0.001 (0.821)	19.45 ± 2.38	18.97 ± 2.42	18.61 ± 2.41 ^abc^	<0.001 (0.728)	26.672	<0.001	0.388
AD	18.29 ± 2.11	17.10 ± 1.90	16.15 ± 1.65 ^abc^	<0.001 (0.884)	18.59 ± 2.16	18.15 ± 2.10	17.79 ± 2.04 ^abc^	<0.001 (0.726)	45.174	<0.001	0.518
PD	17.65 ± 1.48	16.77 ± 1.41	15.75 ± 1.39 ^abc^	<0.001 (0.849)	17.00 ± 1.78	16.74 ± 1.71	16.51 ± 1.76 ^abc^	<0.001 (0.691)	55.507	<0.001	0.575
LD	11.67 ± 1.02	11.02 ± 1.00	10.55 ± 0.95 ^abc^	<0.001 (0.817)	11.75 ± 1.45	11.48 ± 1.37	11.22 ± 1.33 ^abc^	<0.001 (0.736)	18.366	<0.001	0.309

UT: upper trapezius, AD: anterior deltoid, PD: posterior deltoid, LD: latissimus dorsi. ^a^ *p* < 0.05 indicates a significant difference between the baseline and 2 weeks. ^b^ *p* < 0.05, significant difference between the baseline and 4 weeks. ^c^ *p* < 0.05 indicates a significant difference between 2 weeks and 4 weeks.

**Table 3:** Comparisons of shoulder function and quality of life between before and after intervention.

	Manual Therapy				Corticosteroid Injection				Baseline SMD	95% CI ^+^	*p*
	Baseline	2 Weeks	4 Weeks	*p* (η^2^)	Baseline	2 Weeks	4 Weeks	*p* (η^2^)			
Shoulder function											
PSFS	2.53 ± 0.32	5.43 ± 0.48	8.20 ± 0.55 ^abc^	<0.001 (0.987)	2.36 ± 0.36	4.59 ± 0.51	6.68 ± 0.50 ^abc^	<0.001 (0.983)	0.49	1.43 (1.11~1.75)	<0.001
Health-related quality of life											
EQ-5D-5L	0.43 ± 0.04	0.66 ± 0.10	0.80 ± 0.03 ^abc^	<0.001 (0.927)	0.51 ± 0.13	0.75 ± 0.08	0.85 ± 0.03 ^abc^	<0.001 (0.855)	−0.84	−0.018 (−0.038~0.001)	0.070
EQ VAS	25.91 ± 9.46	57.27 ± 8.96	85.45 ± 6.53 ^abc^	<0.001 (0.982)	21.14 ± 7.85	47.73 ± 7.51	72.50 ± 6.12 ^abc^	<0.001 (0.974)	0.55	10.86 (7.65~14.08)	<0.001
GSDS	58.09 ± 15.98	41.23 ± 12.58	27.36 ± 7.56 ^abc^	<0.001 (0.873)	53.23 ± 11.77	37.77 ± 9.57	24.77 ± 7.65 ^abc^	<0.001 (0.942)	0.35	0.44 (−2.57~3.45)	0.767

PSFS: Patient-Specific Functional Scale, EQ-5D-5L: EuroQol-5 Dimensions-5 Level, EQ VAS: EQ Visual Analogue Scale, GSDS: General Sleep Disturbance Scale, CI: confidence interval. SMD denotes the baseline standardized mean difference, calculated as MT minus CI divided by the pooled standard deviation. ^+^ Treatment × baseline interaction model: adjusted difference. ^a^ *p* < 0.05 indicates a significant difference between the baseline and 2 weeks. ^b^ *p* < 0.05, significant difference between the baseline and 4 weeks. ^c^ *p* < 0.05 indicates a significant difference between 2 weeks and 4 weeks.

**Table 4:** Associations of changes in muscle tone and health-related quality of life with shoulder functional recovery (ΔPSFS) (*n* = 44).

Independent Variable	B	SE	β	t	*p*	95% Confidence Interval
Muscle tone						
ΔUT	0.020	0.109	0.023	0.186	0.854	−0.201~0.242
ΔAD	0.051	0.140	0.054	0.363	0.719	−0.333~0.232
ΔPD	0.066	0.158	0.069	0.419	0.678	−0.254~0.387
ΔLD	0.466	0.207	0.268	2.251	0.030	0.047~0.885
Health-related quality of life						
ΔEQ-5D-5L	−0.263	0.873	−0.025	−0.302	0.763	−2.029~1.502
ΔEQ VAS	0.040	0.010	0.372	3.837	<0.001	0.019~0.061
ΔGSDS	−0.006	0.007	−0.070	−0.840	0.406	−0.021~0.009

Δ: change from the baseline to 4 weeks; PSFS: Patient-Specific Functional Scale; UT: upper trapezius; AD: anterior deltoid; PD: posterior deltoid; LD: latissimus dorsi; EQ-5D-5L: EuroQol-5 Dimension-5 Level Index; EQ VAS: EuroQol Visual Analogue Scale.

## Data Availability

The data presented in this study are available on request from the corresponding author due to participant privacy.

## Abbreviations

The following abbreviations are used in this manuscript:

AD	Anterior deltoid
ANOVA	Analysis of variance
CI	Corticosteroid injection
CSP	Chronic shoulder pain
EQ-5D-5L	EuroQol Five-Dimension Five-Level Questionnaire
EQ VAS	EuroQol Visual Analogue Scale
GSDS	General Sleep Disturbance Scale
IFC	Interferential current therapy
IT	Injection therapy
LD	Latissimus dorsi
LLLT	Low-level laser therapy
MT	Manual therapy
PD	Posterior deltoid
PSFS	Patient-Specific Functional Scale
RCT	Randomized controlled trial
UT	Upper trapezius
